# Social and Population Structure in the Ant *Cataglyphis emmae*

**DOI:** 10.1371/journal.pone.0072941

**Published:** 2013-09-09

**Authors:** Michael J. Jowers, Laurianne Leniaud, Xim Cerdá, Samer Alasaad, Stephane Caut, Fernando Amor, Serge Aron, Raphaël R. Boulay

**Affiliations:** 1 Departamento de Etología y Conservación de la Biodiversidad, Estación Biológica de Doñana (EBD-CSIC), Sevilla, Spain; 2 Departmento de Biología Animal, Facultad de Ciencias, Universidad de Granada, Granada, Spain; 3 Department of Evolutionary Biology and Ecology, Université Libre de Bruxelles, Brussels, Belgium; 4 IRBI, Université François Rabelais de Tours, Tours, France; Université Paris 13, France

## Abstract

Dispersal has consequences not only for individual fitness, but also for population dynamics, population genetics and species distribution. Social Hymenoptera show two contrasting colony reproductive strategies, dependent and independent colony foundation modes, and these are often associated to the population structures derived from inter and intra-population gene flow processes conditioned by alternative dispersal strategies. Here we employ microsatellite and mitochondrial markers to investigate the population and social genetic structure and dispersal patterns in the ant *Cataglyphis emmae* at both, local and regional scales. We find that *C. emmae* is monogynous and polyandrous. Lack of detection of any population viscosity and population structure with nuclear markers at the local scale suggests efficient dispersal, in agreement with a lack of inbreeding. Contrasting demographic differences before and during the mating seasons suggest that *C. emmae* workers raise sexuals in peripheric nest chambers to reduce intracolonial conflicts. The high genetic differentiation recovered from the mtDNA haplotypes, together with the significant correlation of such to geographic distance, and presence of new nuclear alleles between areas (valleys) suggest long-term historical isolation between these regions, indicative of limited dispersal at the regional scale. Our findings on the ecological, social and population structure of this species increases our understanding of the patterns and processes involved under independent colony foundation.

## Introduction

Dispersal is a pivotal process with important ecological and evolutionary consequences [1]. In animals, it may be defined as the movement of individuals away from an existing population to a new area where to settle and reproduce. Inbreeding avoidance by long distance gene flow, successful colonization of new habitats and the reduction of local competition for mates and resources are some of the most important fitness benefits of dispersal [2], [3], [4]. However, dispersal is also associated with a number of costs such as the difficulty to find an unoccupied breeding site and a greater exposition to predators [5]. Dispersal does not only affect individual fitness, it also structures local populations and, through the maintenance of long-distance gene flow, it eventually shapes species evolutionary trajectories.

Ants represent interesting model systems to study population genetic consequences of various dispersal strategies at different spatial scales. Many ant species are considered sessile organisms that can occupy the same nests during years while dispersal is mainly determined by the ability of sexuals to fly, often associated to distinctive mating systems [6], [7], [8], [9]. Apart from rare exceptions [10] males always bear wings while queens flying capacity varies greatly between species depending on the colony founding modes. The most ancestral one is independent colony foundation (hereafter, ICF) [11]. This is a solitary dispersal mode where virgin queens bearing long wings (macropterous) leave their mother nest by flight to copulate with one or various males (monoandry vs polyandry). Thereafter they shed their wings and start a new colony on their own. In order to rear their first brood, the founding queens may either forage outside the incipient nest (semi-claustral ICF) or use fat reserves and the energy released by the histolysis of their thoracic musculature (claustral ICF). On the other hand, in the more recently evolved dependent colony foundation (DCF) [9], queen solitary phase has disappeared. The queens, either with small non-functional wings or wingless, either integrate an already established nest, thus leading to highly polygynous nests which progressively split until forming various independent colonies (i.e. colony budding) or leave the natal nest with a worker force to establish a new independent monogynous colony at a walking distance (i.e. colony fission).

DCF is expected to lead to an increase in population structure and promoting an isolation by distance pattern at a local scale [12], [13], [14], [15], [16], [17]. By contrast, the overall advantage of ICF over DCF is longer dispersal distance by flight, up to several kilometers in some species [18]. However, the prolonged exposure to predation and competition by previously established colonies can cause high queen mortality under ICF compared to DCF [8], [19], [20], [21]. The fact that DCF has evolved independently in several species suggests that this mode of colony foundation may be advantageous when associated to specific ecological conditions [9], [22]. A recent study on the ant genus *Rhytidoponera,* which exhibits both reproductive strategies, suggests that the shift from ICF to DCF is gradual and ICF is mostly attributed to advantages of aerial dispersal in open habitats [23].

Although many studies have addressed ant sociogenetics in details, there is limited information on the effect of colony foundation mode on population genetic structure at different spatial scales. The mode of colony founding, dispersal and population genetic structure are generally associated, but they are conceptually different phenomena, so that one should not be deduced from the other. Moreover, other factors such as evolutionary history, past environmental and geographical changes, habitat richness and homogeneity, flight efficiency and other behavioral aspects could have important consequences in the population structure. For example, it was suggested that frequent colony relocations in DCF species could compensate for short dispersal distances leading to population mixing [24]. Moreover, in ICF, even queens with important flying ability may prefer to remain on the same plot rather than attempting the risky colonization of distant areas [25].

The desert ant genus *Cataglyphis* is composed of species with DCF (colony fission: *C. mauritanica, C. niger, C. cursor, C. floricola, C. hispanica*) and ICF (*C. sabulosa*, *C. livida*, *C. bicolor).* Populations of the former are genetically structured while those of the latter show no pattern of isolation by distance [26], [27], [28], [29]. Work on *C. cursor* has shown that its genetic structure at different spatial scales was largely dependent on the sampling distance considered, habitat patchiness and the sex specific dispersal capability. As expected, males contribute more to gene flow at local and regional scales, leading to a pattern of isolation by distance. The limited on foot dispersal of queens (a few meters) results in population viscosity where male gene flow is insufficient to homogenize the female genetic background [30], [31]. Further evidence on the effect of colony foundation is found in *C. mauritanica* (DCF) and *C. bicolor* (ICF). Both species show contrasting genetic mtDNA backgrounds with structured populations in the former but not in the latter [25]. Additionally, the genus *Cataglyphis* is interesting for the great variability of social systems, exhibiting monogyny, polygyny and/or monandry, polyandry, social hybridogenesis and thelythokous parthenogenesis [32], [33], [34], [35].

**Figure 1:**
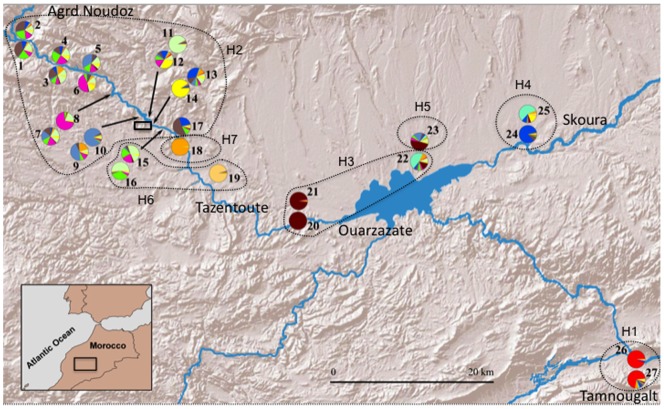
Map of *C. emmae* sampled nest localities across its distributional range (transect) in Morocco near Ouarzazate in 2010. Pie charts are Bayesian estimates of the population structure (log likelihood ln P(D/K). saturation at K = 13) based on microsatellite variation of all 27 sites sampled localities. Dashed areas indicate mitochondrial haplotypes (indicated as H1-7) as inferred from the Medium Joining network. The square by the wadi indicates the location of the fine-scale sampling plot at 1.8 km from Ait Ibourk.

*Cataglyphis emmae* is a common species distributed along presaharian wadis (semi-dry rivers, drying seasonally) of Northern Africa (Fig. 1). Colonies are headed by a single macropterous queen, typical of ICF species. It is the most recent known related species of *C. floricola-tartessica,* a fission-dispersing species complex encountered in Southern Spain ([33]; unpublished data MJJ). Thus, the *C. emmae-tartessica-floricola* group constitutes an interesting model system to test hypotheses on the evolution of dispersal and its consequences for population genetics. In the present study, we addressed the following questions: What is the social structure in *C. emmae* and how does it compare to other species within the genus? Does the observed social structure explain the mating system strategy of this species? How does effective male and female dispersal shape the population structure throughout different scales (local, regional)? Are males responsible for most gene flow at the local scale and is there a correlation of isolation by distance at the local scale?

## Results

### Demography and sociogenetics

We found contrasting demographic differences between nests collected one month before and during the mating period. Nests collected in early April 2011 were composed of 702.5±78.5 workers (mean ± SE; N = 21). All had a queen, small larvae and a few colonies had pupae that were not counted. We found a few large pupae that most likely were sexual offspring but no adult sexuals were found. By contrast, nests excavated in late April-early May 2010 contained significantly less workers (217.9±24.6, N = 17; Wilcoxon test: W = 337.5, *P*<0.001). Because nests were excavated at the beginning of their diurnal activity we did not expect any demographic differences by not collecting the few foraging ants. Furthermore, this small sampling error, difficult to avoid, should not produce any biased results. Moreover, only 25 out of 33 nests excavated during the 2009 and 2010 mating periods (Figure S1) had a queen, while no queen was found in the remaining. All had an abundant brood and 13 contained sexual adults (Queenless nests: 4 nests with males; 20.5±6.22, 3 nests with females; 13.6±10.2, and one nest with males and females; female sex ratio 0.40, Queenright nests: 1 nest with males: 27±0, and 4 nests with females; 1.25±0.5). Interestingly, a significantly higher proportion of queenless than queenright nests contained sexuals (8 of 8 vs 5 of 25, Yates-corrected χ^2^ = 13.07 df = 1, *P* = 0.0003). Numeral sex ratio was not significantly different between queenless (QL) and queenright (QR) nests (0.43±0.18 vs 0.80±0.20, respectively, H_1,13_ = 1.73, p = 0.188).

None of the microsatellite loci showed significant deviations from HWE in 140 tests (20 re-sampled datasets × 7 loci). Of the 300 tests of linkage disequilibrium, only 2 were significant (0.004< *P* <0.05). Given that the two tests could be significant by chance alone, we considered the seven microsatellites to be independent. Between four and 13 alleles were detected per locus with a mean observed heterozygosity *Ho* = 0.47 (range: 0.033–0.664) and a mean expected heterozygosity *He* = 0.36 (range: 0.021–0.503). Genetic descriptive statistics are given in Table 1. During the 2010 field trip we mapped all the nests in a 34×34 m plot near Ait Ibourk. That plot contained 44 nests, the distribution of which was not significantly different from random (489 nests/Ha^−1^; Clark & Evans R = 1.060, *P* = 0.25) (Figure S1). Neighbour nests were separated by 2.5±0.2 m (mean ± SE). Nests were all genetically differentiated and could be considered as independent societies (G-test of differentiation between paired nests: *P*>0.0007 after Bonferroni correction). The fixation index *F_IS_* was significantly different from zero (−0.316±0.026, mean ± SE; 95% CI: −0.422–0.271, t = 12.154 P = 0) suggesting strong outbreeding. *F_ST_* estimate (0.24±0.025; 95% CI: 0.204–0.288 N = 49) indicated high genetic divergence between nests within the plot. Yet it was not significantly different from the overall *F_ST_* calculated over 74 colonies genotyped in the Ait Ibourk area (*F_ST_* = 0.27±0.021; 95% CI: 0.204–0.319; *t* = 0.916; *P* = 0.361).

**Table 1:** Population genetic descriptive statistics of *C. emmae.*

			All population (Nests = 49, N_ind_ = 630)		Transect (Nests = 27, N_ind_ = 146)		Total (Nests = 74, N_ind_ = 643)	
Locus	Allele size range (bp)	N_A_	H_E_	H_O_	H_E_	H_O_	H_E_	H_O_
Ccur11	218 –230	5	0.029	0.037	0.17	0.27	0.083	0.125
Ccur26	96–106	6	0.565	0.775	0.41	0.48	0.503	0.664
Ccur51	195–213	10	0.518	0.657	0.48	0.55	0.509	0.626
Ccur61	217–219	13	0.388	0.477	0.44	0.63	0.403	0.529
Ccur89	114–118	4	0	0	0.05	0.08	0.021	0.033
Ccur99	100–122	12	0.566	0.72	0.46	0.06	0.527	0.675
Ccur100	173–190	7	0.467	0.639	0.5	0.62	0.481	0.638
Overall	-	-	0.362	0.472	0.36	0.46	0.36	0.47

Allele size range (bp); N_A_, number of alleles; N_ind_, number of genotyped individuals; H_E_, expected heterozygosity; H_O_, observed heterozygosity. All population (Nests  = 49) refers to all nests genotyped from 2009 and 2010 (plot scale).

Genetic relatedness between workers and the queen *r_q-w_* = 0.5±0.04 (mean ± SE_jacknife_ N = 24) was not significantly different from 0.5 expected under strict monogyny (*t* = 0, N = 24, *P* = 1.00). Genetic relatedness between nestmate workers *r_w-w_* = 0.43±0.03 (mean ± SE_jacknife_ N = 620) was significantly lower than 0.75 expected between full sibs (*t* = 10.66, P<0.001). However, all typed workers were assigned to the queen present in their nest. When no queen was found in a nest, worker genotypes were compatible with a single inferred queen. Field sample pedigree analyses from workers and queens of 21 nests showed that queens mated with up to 5 males. Absolute and effective patriline numbers were Mp = 3.04±1.04 and Me,p = 2.74±1.09, respectively (Table 2). Ten workers from one nest shared the same paternal alleles supporting the possibility of rare monandry or strong skew between fathers. The average probability of non-detection due to two males bearing the same alleles at all loci was negligible (*P_non-detect_*  = 7.70^−14^). Moreover, queens’ male mates were unrelated to each other (relatedness among a queen male mates: r_m-m_ = 0.01±0.03 (mean ± SE_jackknife_) not significantly different from 0 (*t* = 0.333, N = 63, *P* = 0.74)).

**Table 2:** Queen mating frequency in *C. emmae*.

Colony	nw	M_p_	M_e,p_
E01	20	3	3.29
E02	19	5	3.25
E04	18	2	2.00
E05	19	2	1.83
E06	20	3	2.73
E07	19	3	2.49
E09	11	3	2.54
E13	20	3	2.39
E20	20	4	4.34
E21	20	4	3.41
E22	20	5	5.03
E23	14	2	1.24
E24	19	3	3.25
E31	18	4	3.42
E32	14	3	1.37
E01D	10	1	1.00
E29U	10	3	3.06
E27C	10	4	4.18
E27D	10	2	2.09
E28H	10	3	3.53
E27B	11	2	1.22
Mean ± s.e. (1)	15.8±4.3	3.04±1.04	2.74±1.09

The number of workers (nw) genotyped, the absolute number (M_p_) and effective number (M_e,p_) of matings for each colony.

We investigated the parentage of 41 males collected from five queenless nests. Thirty males collected from four of these nests carried only queen alleles at all loci and were therefore queens' sons. However eleven males from one queenless nest carried at least one non-queen allele at one locus and seven of them carried two non-queen alleles, which were present in the workers. A total of four distinct non-queen genotypes were found. Thus, the males of this nest derived from worker arrhenotokous parthenogenesis.

All 26 alate females encountered in four nests bore a non-queen allele at one locus, which indicated that they had been produced sexually and not through thelytokous parthenogenesis. Consistent with this view, the relatedness between the queens and alate females *r_q-f_* = 0.62±0.08 did not differ significantly from that between queens and workers *r_q-w_* = 0.50±0.04 (*t* = 1.156, *P* = 0.258).

Ten dealated queens were found walking throughout the plot on April, 28^th^ 2010. They were all genotypically different and no other genotyped colony showed the exact same alleles combinations except for one queen showing the same alleles at all loci than two ants sampled in a nest 60 m away from the plot, suggesting a possible origin at that colony.

### Population structure

**Figure 2:**
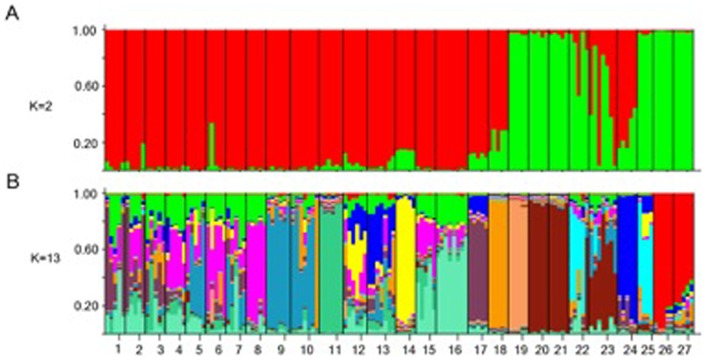
Population structure of *C. emmae* assessed by multilocus genotype clustering by Structure. Each colony is represented by a small vertical bar. (A) population structure following Evanno *et al* 2005 (K = 2). (B) Population structure following the estimated logarithmic probability of the data ln P(D/K) being explained (K = 13). Nest codes (27) start from 1 at Agrd Noudoz (Northwest) and continue increasing numerically to 25 in Skoura (Northeast) and 26–27 at Tamnougalt (Southeast).

Between-nest nuclear genetic differentiation did not correlate significantly with geographic distance at a scale of a few meters (nests sampled within the mapped plot in 2010) or tens of meters (nests sampled in 2009 near Ait Ibourk), indicating no isolation by distance within this population (Figure S2, Table S1). At a larger geographical scale, the results of the Bayesian clustering analysis of multilocus genotypes in Structure analysed in Harvest [36] showed that the log likelihood (ln[P(D/K)] saturated at K = 13 (Fig. 2). An additional *ad hoc* statistic, ΔK, which provides a better predictor of the (K) clusters at the uppermost hierarchical level [37] showed a peak at K = 2 showing a clear genotypic differentiation to the West and to the East of Tazentoute.

**Figure 3:**
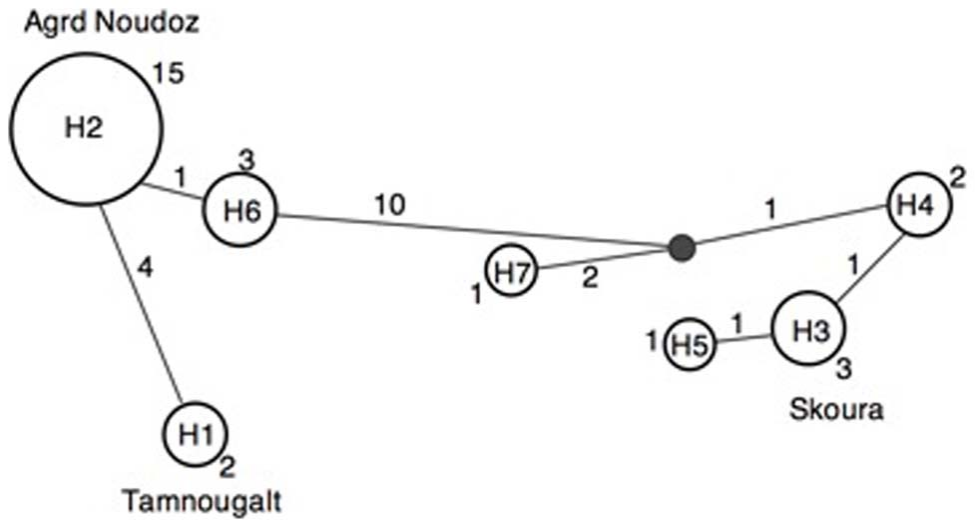
Medium Joining (MJ) network of all seven *C. emmae* haplotypes based in COI partial sequences (622 bp) showing the frequency of each haplotype. The size of the circles corresponds to the haplotype frequency and it is indicated by the numbers by the circles. The small dark colored circle indicates an undetected intermediate haplotype state. Numbers by lines indicate numbers of mutations between haplotypes.

**Figure 4:**
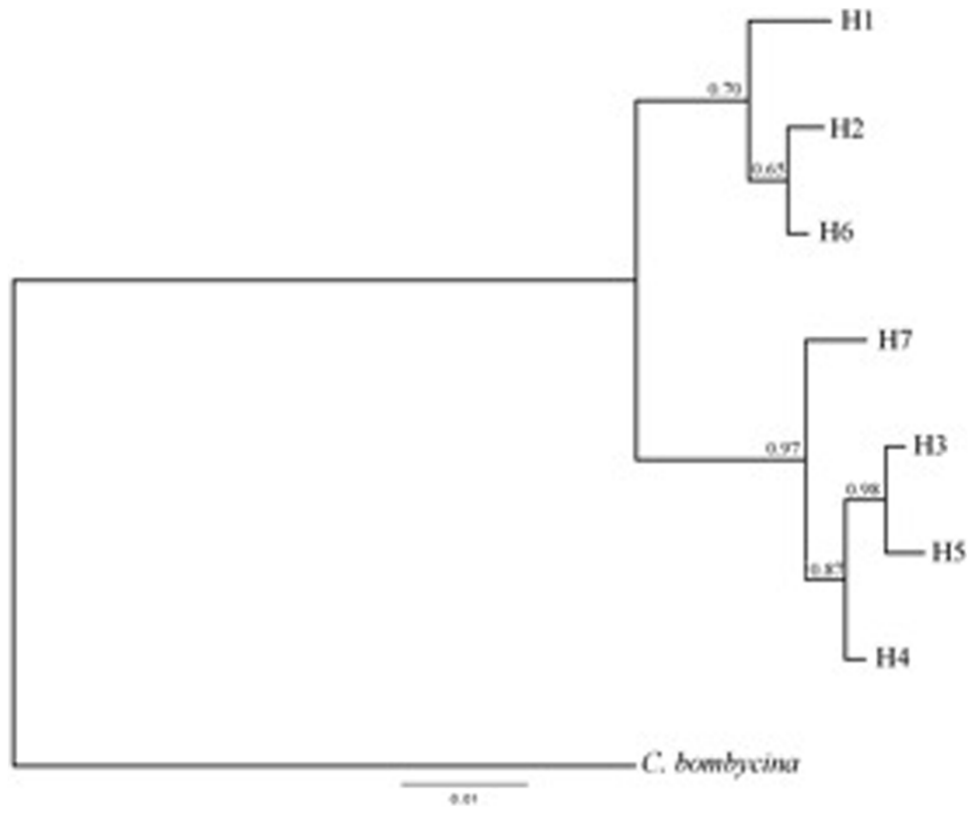
A Bayesian Inference phylogram of all seven *C. emmaé’s* haplotypes obtained from MtDNA COI (622 bp) data. Values by nodes indicate Bayesian Inference posterior probabilities (BPP).

There was a significant positive correlation between geographic distance and mtDNA genetic differentiation at a regional scale along the two wadis that join near the lake of Ouarzazate (Mantel test: *R* = 0.47, *P*<0.001, Figure S1). The mtDNA median joining (MJ) network recovered seven haplotypes (Fig 3) with the highest frequency (N = 15) of H2 from all nests near Agrd Noudoz (North west of Ouarzazate, at down the Atlas mountain slope). Haplotypes clustered in two groups (H1, H2, H6 and H3, H4, H5, H7) though H1 also showed considerable differentiation to all other haplotypes (Fig 1). MtDNA genetic distances within groups were lower (range: 0.16–1.16%) than between groups (range: 1.92–2.57%) (Table 3). Additionally, the highest differences were between H1 from Tamnougalt and H3, H4, H5 and H7 ranging between 2.25–2.57%, indicating a great divergence between them. The best-fitting model for the Bayesian Inference tree was the HKY+I (**−**lnL = 1095.4026, BIC = 2306.5980). The Bayesian Inference phylogram was congruent with the MJ network and recovered two monophyletic clades (H1, H2, H6 and H3, H4, H5, H7). Overall, both methods tended to show an East-West genetic subdivision along the wadis, with exception of H7 grouped with the Eastern haplotypes (Fig 3, 4).

**Table 3:** Nucleotide substitutions (below diagonal) and *p*-uncorrected distances (%) (above diagonal) for each pairwise comparison for all *C. emmae* haplotypes in the transect.

Haplotypes							
	H1	H2	H6	H3	H4	H5	H7
H1	-	0.64	0.8	2.42	2.25	2.57	2.41
H2	4	-	0.16	2	1.92	2.25	2.09
H6	5	1	-	1.92	1.76	2.09	1.92
H3	15	13	12	-	1.16	0.16	0.64
H4	14	12	11	1	-	0.32	0.48
H5	16	14	13	1	2	-	0.8
H7	15	13	12	14	3	5	-

Finally, the genetic differentiation between the two wadis (*F_ST_*) showed marked differences between nuclear and mtDNA data. Hierarchical analyses of molecular variance (Table 4) revealed that the greatest amount of mtDNA variation was between wadis (83–87%) while most of the genetic variation inferred from microsatellites occurred within wadis (79–82%).

**Table 4:** Results of comparative hierarchical AMOVAs for four different populations structures analyzed for mitochondrial and microsatellite data.

Source of variation	MtDNA (COI) (%)	Microsatellites (%)
1	Among populations (all three wadis)	87.1	21.1
	Within populations	12.8	78.8
2	Among populations (Eastern and Western wadis (11 subpopulations))	83.3	17.5
	Within populations	16.6	82.4
3	Among populations (Eastern wadi (3 subpopulations))	66.67*	16.6*
	Within populations	33.33*	83.3*
4	Among populations (Western wadi (8 subpopulations))	29.4*	15.7
	Within populations	70.6*	84.2

All F_ST_ and Φst were highly significant (P≤0.0001) unless indicated (*). The four analyses were: 1; (Western wadi H2, H6, H7 vs Eastern wadi H3, H5, H4 vs Southern wadi H1), 2; Eastern and Western wadis divided into subpopulations (11 subpopulations), 3; Eastern wadi (H3, H4, H5) divided into 3 subpopulations, 4; Western wadi (H2, H6, H7) divided into 8 subpopulations. Subpopulations: sampled points throughout the transect.

## Discussion

The current paper combines field observations and genetic data in order to analyze the sociogenetic structure, mode of dispersal and the population genetic consequences at different spatial scales in a desert ant. The results are congruent with previous morphological studies suggesting that colonies of *C. emmae* are founded independently by a single multiply-mated queen though it may be claustral or semi-claustral. As expected with ICF, microsatellite analysis revealed no evidence of isolation-by-distance at a local spatial scale. Yet, at a regional scale, both nuclear data and mtDNA haplotypes suggest limited gene flow even in absence of clear fragmentation of the habitat. This adds to a series of other studies in the genus *Cataglyphis* giving a better understanding of social evolution and life history traits in this highly diverse group.

### Demography and sociogenetics

Colonies of *C. emmae* contain at most one macropterous queen, but polyandry (M_p_ = 3.0) results in a significant reduction of the genetic relatedness among nestmate workers (*r_w-w_* = 0.43) compared to strict monandry/monogyny. Although monogamy is the ancestral sociogenetic organization of social Hymenopterans, promoting the evolution of eusociality [38], departure from this system is frequent, if not the norm among *Cataglyphis* species. So far, the combination of strict monogyny and monoandry has been reported only in *C. hispanica* [39] whereas levels of polyandry similar or more elevated than in *C. emmae* have been reported in *C. cursor* (M_p_ = 5.6–5.8; [16], [35]), *C. sabulosa* (M_p_ = 2.5; [27]), *C. livida* (M_p_ = 3.8; [28]), *C. savignyi* and *C. niger* (M_p_ = 9.3 and 5.7 respectively, [29], *C. bombycina* and *C. theryi* (M_p_ = 5.7 and 2.5 respectively; [40]). The reason of such a high frequency of polyandry in *Cataglyphis* ants is unknown but it may include increasing parasite resistance, colony efficiency and a reduction of incompatible matings [7], [41], [42], [43], [44].

Although genetic data were compatible with monogyny in all the sampled colonies, no fertile queen was found in 8 out of 33 nests collected during the mating season. Interestingly, adult sexuals were present in all the queenless nests. By contrast, before the mating season, just at the end of the hibernation, all nests contained a queen, more workers and no sexual adults. The fact that the presence of a fertile queen tends to inhibit or delay the development of sexual larvae is relatively common in ants [19], [45], [46]. In the monogynous species *C. iberica*, one colony occupies several nests (polydomy) and adult sexuals are generally found in peripheral queenless nests [47]. However, in *C. emmae*, the high *Fst* values encountered at the local scale, the fact that all nests were different genetic entities and the absence of observation of social transport during our field-work may discard the possibility of polydomy. An alternative explanation is that, though in winter all the workers gather in the royal chamber, in spring sexuals are transported to peripheral chambers connected to the royal chamber through complex and ramified galleries. Enhanced nest complexity may therefore have led us to excavate only a fraction of the colonies. In addition, important forager mortality in spring not replaced by new-born ants may contribute to explain the reduction of worker numbers.

In laboratory conditions, queenless workers of many ant species (including *C. emmae,* MJJ pers. obs.) lay haploid eggs by arrhenotokous parthenogenesis. However, the reality of this phenomenon has rarely been reported in nature. Our data are therefore interesting in showing the males encountered in one out of 5 queenless nests were workers' sons. Worker egg laying may result from a selfish strategy or from a hopeless situation after the death of the mother queen [31]. In contrast to *C. cursor*, *C. hispanica, C. mauritanica* and *C. velox* [34], [35], [39], we did not find any evidence of thelythokous parthenogenesis by *C. emmae* queens or workers.

### Dispersal patterns and phylogeography

The lack of isolation by distance for nuclear markers at a local scale is congruent with important population mixing promoted by ICF. This result is also supported by the observation of dealated queens in our study plot originating from nests located at least 60 m away. This distance is much greater than the average distance of colony fission in the related species *C. floricola* (7.7±0.9 m; [48]). It is not known if the observed dealated queens were searching for a nesting site or if they were foraging which would imply semi-claustral independent colony founding.

At a larger geographical scale, the analysis of mtDNA polymorphism suggests queen movement between neighbor populations is limited. However, several mtDNA haplotypes common in one area were absent in all others with only one mtDNA haplotype (H3) shared between the two wadis near the Ouarzazate lake. The low mtDNA genetic diversity observed at a local scale suggests recent and rare maternal lineage colonization events as well as efficient female dispersal within localities. Evidence of this can be seen in the last 30 km of *C. emmae* distribution up to Agrd Noudoz, where the Atlas Mountains act as a geographical barrier and prevent further migration in that direction (Fig 1).

*C. emmae* is exclusively distributed along the wadis. The high level of mtDNA differentiation between Tamnougalt and the other localities is likely due to a lack of gene flow provoked by arid mountains where we were unable to locate *C. emmae* while other *Cataglyphis* species were present (e.g. group *albicans*, MJJ pers. obs.) during our census (Fig 1). However, the opposition between the other two clusters (H2, H6 and H3, H4, H5, H7) cannot be explained by evident habitat fragmentation of the wadis. We propose three hypotheses, not necessarily mutually exclusive to explain this pattern: First, aerial dispersal is likely to occur through the wadis aided by wind currents. Northwestern winds between Agrz Noudoz to Tarentoute fit the observed pattern of lack of haplotype diversity in the wadi and the higher haplotypic diversity towards the Quarzazate lake, where the wind currents flow to. The trade winds, flowing in a Northeastern direction, are predominant between the Skoura-Quarzazate wadi and thus are likely to contribute to the mixing of haplotypes near the Quarzazate lake area where Northwestern and Northeastern winds meet. Second, human settlements and environmental disturbance through agricultural land today differentiate the Western, more arid, and Eastern, greener, agricultural pastures. This pattern of environmental disturbance between East and West is reflected by the two highly genetically differentiated groups constituted by H2, H6, (East) and H3, H4, H5 and H7 (West) (Fig. 2) suggesting important past historical events resulting in genetic isolation between localities. This is apparent with the grouping of H1 with the Western wadi, likely the result of past historical events in the area and genetic isolation at such locality (Fig. 1). Similarly, the presence of agricultural lands has been suggested to have an impact in the habitat colonization in *C. mauritanica* and *C. bicolor* [29]. Third, despite the fact that *C. emmae* queens seem to bear large wings suitable for flight (although we never observed one flying), there is growing evidence that the physical or mechanical ability to fly is not the primary cause limiting female dispersal and that ethological factors may be more important for this purpose [12]. Hence, abiotic (eg., humidity, temperature), and biotic factors such as ecological constrains to foraging efficiency, resource availability, mate preferences, competition and predation may play important roles preventing a more female effective dispersal, which are likely dependent on the habitats within and in proximity to the wadis.

The contrasting genetic differentiation between the mtDNA and nuclear markers within wadis could be an indication of higher within-population gene flow by males than by queens. This is also evident from the many nuclear alleles unique to regions and/or localities [30]. Male-biased dispersal would be in agreement with field observations of other *Cataglyphis* ICF species (*C. livida, C. sabulosa*) where queen dispersal occurs after mating. Males fly in swarms on relatively long distances to copulate with queens near their mother nest entrance [27], [28]. Queens then fly away and by so doing participate to secondary dispersal of male genes through the sperm stored in their spermatheca. Similarly to *Cataglyphis*, male-biased dispersal was also shown in other ICF species (e.g., *Solenopsis invicta*, *Formica exsecta*), in which queens bear long wings but contribute less than males to within population gene flow [49], [50]. However, the comparison of biparentally-inherited nuclear and mtDNA maternally-inherited markers should be treated with caution because mtDNA differentiation may be higher than nuclear due to its smaller effective population size [51] and larger susceptibility to genetic drift [52]. Furthermore, polyandry also contributes to males having a larger effective dispersal population size.

*Cataglyphis emmae* population structure is similar to those of other monogynous and polyandrous ICF *Cataglyphis* species like *C. sabulosa*, *C. livida, C. bombycina, C. theryi* [27], [28], [39]. The five species have high Fst values (0.24, 0.27. 0.21, 0.17 and 0.28 respectively) even at a population scale, with lack of inbreeding and no isolation by distance. The finding of *C. emmae* same mtDNA haplotypes clusters (eg., H2 and H3) many kilometers distant have also been observed in *C. bicolor* (ICF) but not in DCF species such as *C. mauritanica* and *C. cursor* [26], [30]. We hypothesize that *C. emmae* population structure maybe associated to their habitat requirement and wind current migration through the wadis, following a directional dispersal pattern. Future comparisons of *C. emmae* with *C. floricola-tartessica* species complex is likely to give better insight into the evolutionary and the ecological consequences of divers mating systems and how and what factors may influence the transition from ICF (*C. emmae*) to DCF (*C. floricola-tartessica*).

## Methods

### Ethics statement

No ethics guidelines are required or established by the Spanish or Moroccan authorities to work on ants. The project licence to work in Morocco was granted by the AECID (Agencia Española de Cooperación Internacional y Desarrollo) by the Secretario General Juan Pablo de Laiglesia (Project Number A4774/06). Work was carried out on Moroccan public and non-protected land and the model ant was not a protected species.

### Field sampling

All sampling was conducted in the South of the Atlas Mountains (Morocco). In order to investigate *C. emmae* sociogenetics and population genetic structures, we first excavated 16 nests in late April-early May 2009 (during the mating period) in a zone about 1.8 km South of the locality of Ait Ibourk (Fig. 1). These nests were separated by 10 to 100 m. For each of them, we recorded the number of queens and sexuals (males and alate females) and collected samples of 8–20 workers for genotyping.

A finer scale sampling was conducted at the same site in late April 2010. To that end, we delimited a 34×34 m (1166 m^2^) plot at about 50 m from the 2009 site. All nests within the plot were mapped to estimate nest density and spatial distribution. Active nests were located by offering a piece of biscuit to workers and following them to the nest. The map was considered complete when no new nest was found and all foragers reached previously marked nests. Sinclair's (1985) correction of Clark & Evans (1954) statistics [53], [54] was used to estimate the nearest neighbour distance and to analyse nest local distribution patterns as explained in [47]. Seventeen nests located in the plot were excavated and their full demography (queens, sexuals and workers) was recorded. Samples of 11 of these nests were used for genotyping. Worker samples were also taken from the entrance of 22 other nests within the plot. On the 28th April 2010, 10 dealated queens that were walking in the mapped plot were also collected for genotyping. Finally, an additional 21 colonies were excavated in mid April 2011 (approximately one month before the mating period) in the same plot or close to it for complementary demographic analyses. The numerical sex ratio (*nSR*) was calculated for each nest containing at leat one adult sexual as the proportion of female sexuals over the total number of sexauls. nSR was compared between queenless (QL) and queenright (QR) nests (2009–2010) with a Kruskal-Wallis test (hereafter KW).

To assess the genetic structure at a regional scale we sampled 27 *C. emmae* (one worker per colony) colonies from 14 sites along a 150 km transect (Fig. 1). This transect covered most of the known distributional range of *C. emmae* in the region and followed two wadis, East and West from Ouarzazate. The most Northwestern sampled nests were 1 km South from Agrd Noudoz (59 km from the city of Ouarzazate following the road), before the Atlas Mountains and the most Eastern nests (31 km from Ouarzazate) were just 3 km East from Skoura. Two colonies were sampled from an additional site located 54 km Southeast from Ouarzazate, 3 km before the locality of Tamnougalt, possibly the last locality of *C. emmae* before the dune formations of the Saharan desert. At all localities samples were taken from two nests less than 50 m apart, with the only exception of one single nest found at locality number 19 (Fig 1). Sampling was limited due to the low numbers of nests found at the different localities throughout the transect area.

### Genetic analyses

A total of 663 workers and sexuals from 74 colonies were genotyped (average  = 9.0 workers per colony). DNA was extracted from the brain and surrounding musculature. This soft tissue proved more suitable for efficient PCR amplification than other body parts containing quitine, such as legs and thorax, which inhibited the PCR reactions. DNA extraction followed the HotShot method [55] and was then stored at −20°C.

Seven microsatellite markers developed for *C. cursor* (Ccur11, Ccur 26, Ccur 51, Ccur 61, Ccur 89, Ccur 99 and Ccur 100; [35]) were used to study nuclear polymorphisms in *C. emmae*. Polymerase chain reactions (PCR) were carried out in pairs (duplex reaction) or individually. Each 20 µl PCR volume contained approx. 50 ng DNA, 200 µM of each dNTP, 0.15 µM of each primer, 2 µl Buffer 10X, µl Mgcl_2_ and 0.1 unit of taq polymerase (QIAGEN). The thermal cycle profile was as follows: an initial denaturation step of 2 min at 94°C; 35 cycles of denaturation at 30 s at 94°C, annealing for 30 s at 52°C and extension for 45 s at 72°C; and a final extension for 5 min at 72°C. Following the PCR reactions, excess primers and dNTPs were removed using enzymatic reaction of *E. coli* Exonuclease I, Antartic phosphatase and Antartic phosphatase buffer (all New England Biolabs). Sequencing was carried out in both directions using the BigDye^®^ Terminator v1.1 cycle sequencing kit (Applied Biosystems) according to the manufacturer’s instructions. Labelled fragments were resolved on an automated A3130*xl* genetic analyzer (Applied Biosystems). Incomplete terminal sequences and PCR primers were removed**.** Control for genotyping errors due to null alleles and allele drop-outs was performed with Micro-checker [56]. Linkage desiquilibrium, Hardy-Weinberg equilibrium tests and basic statistics were performed in GENEPOP ON THE WEB [57]. Due to the strong family structure present in colonies, genotypes within colonies were not independent. Thus, only a single individual per colony was used for these tests. A re-sampling procedure was performed in which a single individual from each colony was selected at random for a total of 20 replications according to [58].

Mitochondrial DNA variation was assessed from all colonies sampled from the regional scale transect (Fig. 1). The primers Cflor (L) 5′-TGCAGGAACAGGATGAACAA-3′ and Cflor (R) 5′-TGGCCCATCATAAAGATGAA-3′ amplified approximately a 660 base pair fraction of the cytochrome oxidase subunit (COI, genbank accessions: JQ801346-72). PCR conditions were exactly as those described for the nuclear markers. Templates were sequenced on both strands, and the complementary reads were used to resolve rare, ambiguous base-calls in Sequencher v.4.9. After removing PCR primers and incomplete terminal sequences, 622 base pairs were available for analyses. All nucleotide sequences could be aligned without gaps, when translated into amino acids using the invertebrate mitochondrial code, stop codons were absent. Sequences were aligned in Seaview v.4.2.11 [59] under ClustalW2 [60] default settings. Nucleotide substitutions and *p*-uncorrected distances (%) analyses were performed with PAUP*4.b.10 [61].

The most appropriate substitution model for the Bayesian Inference (BI) analyses was determined by the Bayesian Information Criterion (BIC) in jModeltest v.0.1.1 [62]. The tree was constructed using the Bayesian Inference (BI) optimality criteria under the best fitting model (HKY+I). MrBayes v.3.1.2 [63] was used with default priors and Markov chain settings, and with random starting trees. Each run consisted of four chains of 10,000,000 generations, sampled each 10,000 generations for a total of 750 trees. A plateau was reached after few generations with 25% (250 trees) of the trees resulting from the analyses discarded as “burn in”. In order to assess the relationship between both morphs, an outgroup was choosen to root the phylogenetic tree. *C. bombycina*, from the *C. bombycina* group, and basal to the *C. emmae* group [64] was chosen for this purpose.

### Sociogenetics

Descriptive genetic statistics (*i.e.,* the number of alleles, allele frequencies, observed heterozygosity and expected heterozygosity) and Wright’s *F*-statistics (inferred from individuals within nests) were computed with FSTAT [65] and GENEPOP ON THE WEB [57]. We tested the genetic entity of nests that were close (>0.5m) and could therefore be part of the same colony by genotyping workers at all collection points (nest entrances) and comparing them using a likelihood (G) based test differentiation in GENEPOP ON THE WEB. The overall significance was determined using Fishers’s combined probability test. A Bonferroni correction was applied to account for multiple comparisons. Samples were considered from different colonies if genotypic differentiation was statistically significant (α<0.0007) after the Bonferroni correction.

Relatedness coefficients *r* were estimated in Relatedness (v 5.0.8) according to [66]. All colonies were equally weighted and standard errors were obtained by jackknifing over colonies. Only (N = 36) colonies with at least eight workers were included in the analyses (total sample size for relatedness analyses: N = 620 individuals including 9 and 62 inferred queens and queenś mates respectively). The total of 15 queens were genotyped. *C. emmae* being strictly monogynous (see Results), when no queen was found during excavation, the genotype of the presumed queen was reconstructed from worker genotypes (N = 9 queens).

Individuals were assigned as belonging to different matrilines if they did not share an allele with the (presumed) queen at least at one locus. Assignment of individuals to matrilines was confirmed with the maximum-likelihood methods implemented in the program COLONY 1.2 using of 21 colonies with a minimum of 10 workers (average  = 15.8) were run in COLONY [67].

The absolute number of matings per queen (M_p,_ the minimum number of males inferred from worker genotypes) was estimated on the basis of mother-offspring allele combinations. Because males may contribute unequally to the offspring, we estimated the effective mating frequency (M_e, p_) following [68]:
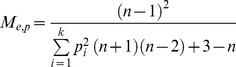



where *n* is the total number of offspring of a queen, *k* is the number of males, and *p* is the proportional contribution to the brood to the *i* ^th^ male. This estimator has the advantage of being unbiased by the relative contribution of each male and gives a lower variance than other estimators. The effective number of patrilines equals the absolute mating frequency when all males contribute equally. Because two males may bear the same alleles at all the loci studied, we estimated the non-detection error by calculating the probability that two mates bear the same alleles using [69]:
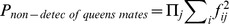



Where *f_ij_* is the allelic frequency at the population level of the *i* allele at the *j* locus.

Worker parentage was investigated by comparing males to queen genotypes. A total of 41 males were genotyped from five colonies (N = 5, 8.4±4.6 males per nest). Queen sons must carry a queen derived allele at a loci, and as a group, they should not display more than two alleles at a single loci. Workers' sons can carry with the same probability a mother or father derived allele. A male with non-queen alleles is a workeŕs son. Nevertheless, worker sons may carry queen alleles at all loci by random chance. To account for this probability of non-detection, we estimated it following [70]:
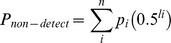
Where *n* is the number of patrilines in the colony, *pi*, is the proportional contribution of the *ith* father to the brood and *li* is the number of informative loci analysed at the *ith* patriline.

Examination of *C. emmae* asexual reproduction for queen production, as seen in *C. cursor* [38], was investigated by comparison of the queen pedigree to that of her daughters. A total of 26 alate females belonging to four nests were examined. Females with alleles identical to that of the mother could potentially be the result of thelytokous parthenogenesis.

### Population structure

The pattern of isolation by distance was tested by plotting (F_ST_/ (1-F_ST_)) coefficients between pairs of colonies against the logarithm (ln) of geographical distances [71], [72]. The significance of Spearman rank correlation coefficient (two-tailed) between genetic differentiation and geographical distance was assessed using Mantel tests with 10,000 permutations (GENEPOP ON THE WEB) or in IBDWS (Isolation by distance web service) v.3.16 [73].

Haplotype frequencies and reduction were estimated using a Median joining (MJ) network constructed with Network 4.5 [74] with default settings.

The Bayesian clustering software Structure 2.1 [75] was also used to infer the number of populations (K) independent of spatial sampling. Analyses were performed using the admixture model with correlated allele frequencies in twenty independent runs from K = 1 to K = 20, with a burn-in of 100,000 iterations followed by another 100,0000 iterations. Selection of K was determined using two methods which were run in Harvest v. 0.6.1 [36]: (i) by plotting the negative log-likelihoods [(ln P(D)] versus K, and (ii) using the ΔK method described in [37]. All statistics were performed in Microsoft Excel or SPSS v.19 and two tailed t-tests were performed to assess statistical significance between means when possible.

The extent of geographical structuring of genetic variation between *C. emmae* from all sampled Wadis (East and West from Ouarzazate and at the Tamnougalt locality) was evaluated by Fst and Φst statistics using the analysis of molecular variance (AMOVA) in ARLEQUIN [76], [77]. The significance of variance components and *F*-statistics were assessed by permutations (10,000) of the data sets.

## Supporting Information

Figure S1 **Plots showing the ants nests sampled starting from a larger (2009 and 2010 nest sampling) to a smaller scale (only 2010 sampling).** The vertices indicate the measured plot area from where nests were sampled. Lines delimit different independent approximate transects to assess genetic divergence by distance (mantel tests) and the circle delimits a mantel test performed for all nests in such circle. Line 1; (5 nests), line 2; (6 nests), line 3; (6 nests); lower section of genotyped nests from 2009 (12 nests in circled area); all nests in mapped plot (31 nests), all nests combined (49 nests). See Table S1 for mantel tests statistics.
(JPG)

Figure S2 **Relationship between geographical distance and mitochondrial genetic differentiation between nests for the transect, estimated as F_ST_/(1-F_ST_).** The correlation is highly significant (R = 0.47, p<0.001).
(JPG)

Table S1 **Mantel tests analysed and statistics for genotyped nests from nuclear data (microsatellites).** All results were non significant. See Figure S1 for details of transects used to test possible correlation of genetic divergence and geographic distance at a local scale.
(DOC)
