# Supplementary figures and images for: Social and Population Structure in the Ant Cataglyphis emmae
Source: PLoS One. 2013 Sep 9;8(9):e72941. doi: 10.1371/journal.pone.0072941 (PMC3767659; doi:10.1371/journal.pone.0072941)

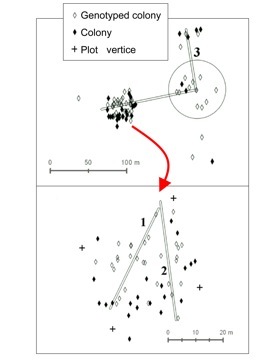

Supplement: Figure S1 — Plots showing the ants nests sampled starting from a larger (2009 and 2010 nest sampling) to a smaller scale (only 2010 sampling). The vertices indicate the measured plot area from where nests were sampled. Lines delimit different independent approximate transects to assess genetic divergence by distance (mantel tests) and the circle delimits a mantel test performed for all nests in such circle. Line 1; (5 nests), line 2; (6 nests), line 3; (6 nests); lower section of genotyped nests from 2009 (12 nests in circled area); all nests in mapped plot (31 nests), all nests combined (49 nests). See Table S1 for mantel tests statistics. (JPG) [file pone.0072941.s001.jpg]

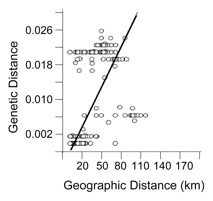

Supplement: Figure S2 — Relationship between geographical distance and mitochondrial genetic differentiation between nests for the transect, estimated as FST/(1-FST). The correlation is highly significant (R = 0.47, p<0.001). (JPG) [file pone.0072941.s002.jpg]
