# Supplementary material for: Social and Population Structure in the Ant Cataglyphis emmae
Source: PLoS One. 2013 Sep 9;8(9):e72941. doi: 10.1371/journal.pone.0072941 (PMC3767659; doi:10.1371/journal.pone.0072941)
Supplement: Table S1 — Mantel tests analysed and statistics for genotyped nests from nuclear data (microsatellites). All results were non significant. See Figure S1 for details of transects used to test possible correlation of genetic divergence and geographic distance at a local scale. (DOC) [file pone.0072941.s003.doc]

| Lines and areas | Spearman´s (two tailed) rank correlation coefficient | |
| --- | --- | --- |
|  | *r* (p) | *Spearman´s rho* (p) |
| LINE 1 (N=6) | -0.389 | -0.493 |
| LINE 2 (N=5) | 0.205 | 0.467 |
| LINE 3 (4 nests ina rea 2009 and 2 in area 2010) | 0.353 | 0.221 |
| 2009 (N=12) | -0.036 | -0.083 |
| Mapped plot (N=31) | -0.045 | -0.057 |
| All nests (N=49) | 0.035 | 0.075 |
